# The evolution of the immune system of bees is defined by conservation, expansions, and losses

**DOI:** 10.1186/s12915-026-02564-0

**Published:** 2026-03-10

**Authors:** Hongfei Xu, Ina Köhler, Thomas J. Colgan

**Affiliations:** 1 Institute of Organismic and Molecular Evolution, Johannes Gutenberg University Mainz, Hanns-Dieter-Hüsch-Weg 15, Mainz, 55128 Germany; 2 Institute of Quantitative and Computational Biosciences (IQCB), Johannes Gutenberg University Mainz, Hanns-Dieter-Hüsch-Weg 15, 55128 Mainz, Germany

**Keywords:** Bees, Immunity, Comparative genomic analysis, Gene duplication, Antimicrobial peptides

## Abstract

**Background:**

Insect pollinators, such as bees, provide essential ecosystem services yet face increasing environmental challenges, including pathogens, which can negatively impact host fitness. Central to host defences are immune genes and their products but for many bee species, our understanding of the conservation of the immune gene repertoire, as well as mechanisms that allow for functional diversity, is restricted to a few species.

**Results:**

Here, we perform a pan-clade examination of the canonical immune genes and associated functional gene groups found across 70 bee species, representing six of the seven extant families. We show a high level of conservation of immune genes with all major immune gene groups represented, with elevated copy number variation found in CLIP-domain serine proteases, serpins, and small RNA regulatory proteins across bee species. Using the buff-tailed bumblebee *Bombus terrestris*, we further show that increases in immune-related gene group size are generally associated with increased nucleotide diversity, and transcriptional divergence among group members suggestive that group size, through mechanisms such as gene duplication, may allow for structural and functional diversity across immune components. However, we find that this pattern is non-linear indicating that gene group expansion is constrained. We also find lineage-specific losses of antimicrobial peptides highlighting that certain immune components may be dispensable or compensated by other elements.

**Conclusions:**

Our analyses show that the genetic components of bee immunity are largely conserved, with duplication and loss highlighted as mechanisms that shape immune diversity, which, collectively, has implications for understanding resilience of bee species to increasing pathogenic threats.

**Supplementary Information:**

The online version contains supplementary material available at 10.1186/s12915-026-02564-0.

## Background

Pathogens covet different components of a host’s wealth. Through the evolution of traits and strategies, pathogens can exploit their host, which can result in extensive host morbidity and mortality. Given the fitness costs associated with infection and associated disease, hosts evolve counter-adaptations, which range from the molecular to the organismal level, to avoid, mitigate, or reduce infection [1–3]. However, if pathogens successfully encounter a host and overcome its physical and chemical barriers, the host immune system represents a formidable last line of defence, acting to prevent the establishment of infection and the progression to disease [4]. The immune system is a highly coordinated network of diverse components, encoded by a repertoire of genes, that function in the recognition of foreign entities, activation of immune signalling pathways, and generate effector molecules, to directly target pathogens or reduce their spread [5, 6]. A fundamental aspect of the immune system is also its ability to distinguish between self and non-self components, serving a dual function in the detection of pathogenic threats, while also avoiding self-harm and inappropriate responses against harmless substances [7].

Insects demonstrate remarkable diversity in terms of immune gene composition, driven by evolutionary innovations and functional specialisation. Unlike vertebrates, insects lack the adaptive immune response, yet have evolved an innate immune system, consisting of cellular and humoral components that act to defend hosts against pathogens [8, 9]. The core of the insect innate immune system is reliant on signalling pathways, such as Toll, Immune Deficiency (IMD), and JAK/STAT, yet genes encoding for recognition (e.g., peptidoglycan recognition proteins, Gram-negative binding proteins), effector (e.g., antimicrobial peptides), and regulatory molecules (e.g., serine protease inhibitors, autophagy) show striking evolutionary dynamism [10].

Given the critical role of the immune system in defending against pathogens, immune genes across many taxa are often considered fast-evolving, facilitating adaptations or counteradaptations in host–pathogen interactions [11–13]. Innovation within the immune system can also be facilitated through gene duplication events, which can facilitate structural and functional diversification of immune genes, allowing for the evolution of expanded gene families that aid in the response to diverse pathogens [14]. This is particularly true for expanded gene families, such as the CLIP-domain serine proteases and serine protease inhibitors (serpins), which perform roles in the activation and regulation of key insect immune defences, such as the melanin-producing prophenoloxidase (proPO) cascade [15], and immune pathways [16, 17]. However, duplication of copy number may also lead to functional redundancy leading to the rapid gene turnover [18] and copy losses through either neutral or adaptive processes [19, 20]. Immune system diversity and innovation can also be aided through the functional convergence of non-paralogous genes through co-option and recruitment to perform a coordinated or shared function as has been described for small RNA regulatory proteins, which perform important defensive roles against viruses [21] and transposable elements [22]. Therefore, through examination of gene duplication and expansion of gene group size, we can identify where selection may act to increase innovation within the immune system.

A group that has greatly contributed to our understanding of the evolution and expression of the insect immune system are bees (Order Hymenoptera: Clade Anthophila), which comprise approximately 20,000 species worldwide, classified into seven extant families, and are found on all continents except Antarctica [23]. Bees are essential for maintaining crop yields that depend on animal-mediated pollination, as well as supporting wildflower diversity and overall ecosystem stability [24, 25]. Like other declining insect species, recent regional reductions in bee populations have raised concerns over the continued provision of vital pollination services [26, 27]. Both abiotic and biotic factors contribute to these declines, with pathogens identified among the primary drivers [28]. The risks posed by pathogens are underscored by the devastating impact of virus-transmitting *Varroa* mites on global honeybee populations [29], emphasising the need to understand disease dynamics, as well as the capacity of bee species to respond. Similarly, the increase in commercial trade of social bees, especially in regions where they are non-native [30], can facilitate the introduction of new pathogens or more virulent strains to wild bees, which may act as non-target hosts [31–34]. Given the concern over the impact of emerging and novel strains, improving our understanding of the immune potential of bees is crucial.

Given the importance of the immunity in bees, the genomic bases of their immune system were first described in the western honeybee, *Apis mellifera* [35, 36]. At the time, comparative genomics of the immune gene repertoire was largely limited to a few insect species. Initial characterisation of canonical immune genes revealed that, while the honeybee genome contained representatives of all core insect immune pathways and gene families, it had reduced numbers of immune genes compared to other insect species, such as *Drosophila melanogaster* and *Anopheles gambiae* [35]. Subsequent genome-based analyses of other bee species, including both social and solitary species, revealed similar numbers of immune genes [37, 38], although more recent analyses suggest that the immune repertoire in bees may be larger than previously thought [39, 40]. Indeed, microevolutionary analyses of immune genes indicate that different components of canonical immune genes of bees are evolving under varying selective pressures, including evidence of functional loss that suggests redundancy within certain gene families [41]. While structural and associated functional redundancy can lead to pseudogenisation and eventual gene loss [19], gene duplication also serves as an important mechanism for evolutionary innovations [42]. Gene duplication can lead to the evolution of new functions (neofunctionalisation), the subdivision of ancestral functions (subfunctionalisation), and may also help resolve intragenomic conflict [43, 44]. Evidence of lineage-specific expansions in certain bee species further highlights its role in shaping immune gene diversity [37]. However, to date, despite bees comprising a taxon-rich group, and the increasing availability of high-quality bee genome assemblies, comparative genomics of immune genes has been, thus far, restricted to a few species and families [45], limiting our understanding of the immune repertoire and its variation found across bees as a group.

Using a comparative genomic approach, involving seventy species from six extant bee families, we conducted a homology-based analysis to identify the presence and conservation of canonical immune gene groups within the bee (Anthophila) lineage. Second, we examined copy number variation within and across prominent immune-related functional gene groups (here, defined as a group of genes that either share an evolutionary history or function in a shared biological role, and may consist of one or more gene families), as through gene duplication events, larger gene groups may display greater structural and functional variation in terms of greater interspecific divergence, and intraspecific polymorphism. Lastly, using publicly available transcriptomic datasets for the bumblebee *Bombus terrestris*, we investigated whether larger functional gene groups have greater signatures of transcriptional specialisation, which may be facilitated or promoted as a consequence of such groups expanding their copy number.

## Results

### Main immune-related gene groups are conserved across bee species

**Fig. 1:**
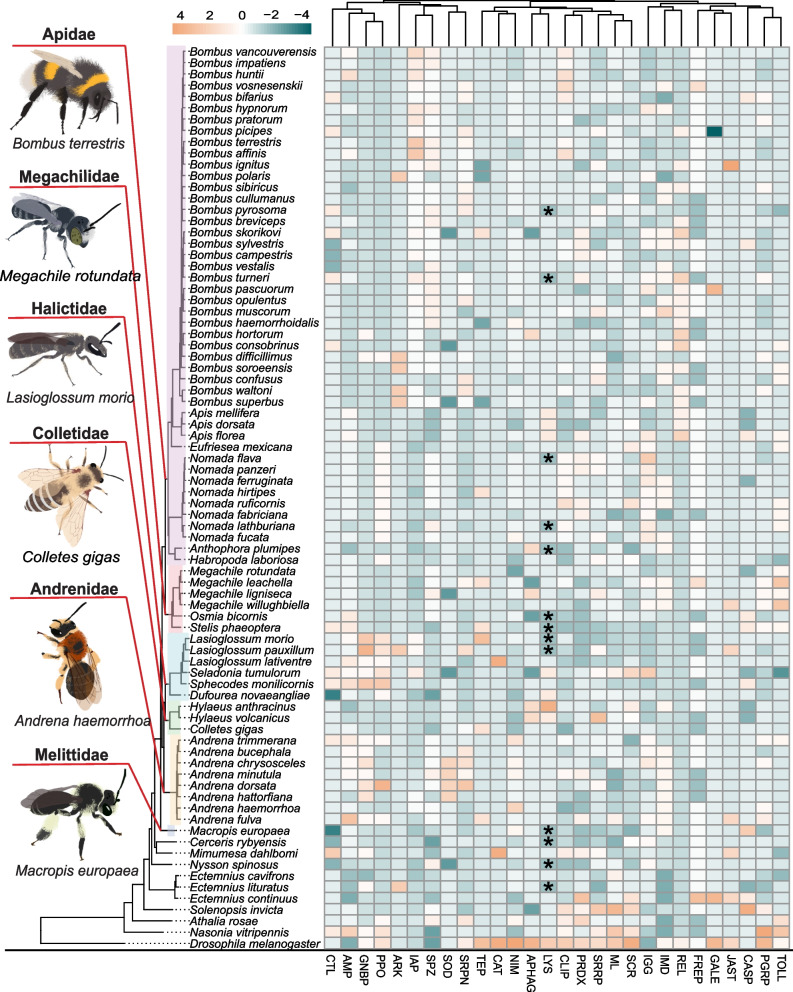
Canonical immune-related functional gene groups are largely conserved in bee species. A heatmap displaying the z-score scaled gene numbers per species for each canonical immune-related functional gene group. A phylogenetic tree informed by our homology-based analyses of the predicted proteomes for 80 insect species, including representative members of six of the extant bee families (Apidae = light purple; Megachilidae = pink; Halictidae = light blue; Colletidae = light green; Andrenidae = light yellow; Melittidae = grey), is also provided. Individual families of bees are represented by individual colours while an asterisk (*) indicates where a gene group was not detected based on our initial homology-based analysis. Raw gene counts for each species are provided in Additional file 1: Table S2. (Abbreviations: AMP = Antimicrobial peptide; APHAG = Autophagy; ARK = Death-associated APAF1-related killer; CASP = Caspase; CAT = Catalase; CLIP = CLIP-domain serine protease; CTL = C-type lectin; FREP = Fibrinogen-like; GALE = Galectin; GNBP = Gram-negative binding protein/Beta-glucan recognition protein; IAP = IAP repeat, inhibitor of apoptosis domain; IGG = Immunoglobulin; IMD = Imd pathway; JAST = JAKSTAT; LYS = Lysozyme; ML = MD-2-related lipid recognition; NIM = NIMROD; PGRP = Peptidoglycan recognition protein; PPO = Prophenoloxidase; PRDX = Peroxidase; REL = Relish; SCR = Scavenger receptor; SOD = Superoxide dismutase; SPZ = Spaetzle; SRPN = Serine protease inhibitor; SRRP = Small RNA regulatory pathway; TEP = Thioester-containing protein; TOLL = Toll genes, Toll pathway)

To determine the conservation of the bee immune system at the genomic level, we first performed a pan-clade homology-based analysis using the entire predicted proteomes of 70 bee species, including representatives from six of the seven extant bee families, as well as closely related hymenopteran outgroups (*n* = 9 species; Additional file 1: Table S1). Through this analysis, we constructed orthogroups, each comprising putative homologous proteins shared among the focal insect species. To evaluate the conservation of putative immune genes across representative bee species, we focussed on a subset of orthogroups (*n* = 167) containing predicted protein sequences of canonical immune genes, previously described and classified into 28 immune-related functional gene groups in the large earth or buff-tailed bumblebee *Bombus terrestris* [37, 38] (Full list of gene groups are in Additional file 1: Table S2). Through this homology-based analysis, which relied on protein-to-protein searches, we found that all bee species possess at least one member of each immune-related functional gene group (Fig. 1; Additional file 1: Tables S2 and S3), with the exception of the effector molecules, lysozymes (LYS), which were not detected in 13 species. To further investigate this absence, we performed a tBLASTn search of predicted lysozyme proteins from other bee species against translated nucleotide sequences derived from each reference genome assembly, revealing putative coding sites within species lacking predicted proteins. We further examined whether these matched genomic regions were predicted to code for proteins containing conserved functional domains identified within known lysozyme proteins. Using InterProScan [46], we found that all examined bee genome assemblies contained evidence of coding for a protein containing a predicted glycoside hydrolase family 22 domain (IPR001916), which is a common domain found in lysozymes [47] (Additional file 1: Tables S4 and S5). Collectively, these comparisons indicate that the core immune-related functional gene groups, encompassing key pathogen recognition molecules, major immune pathways, effector molecules, and regulatory components, are conserved across bee species (Fig. 1).

### Increased copy number variation with gene group size

**Fig. 2:**
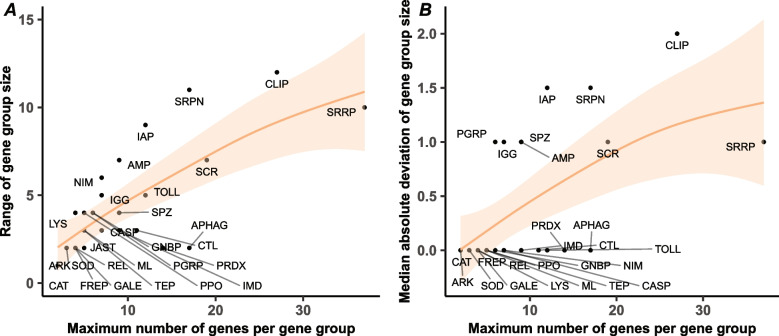
Larger immune-related functional gene groups show greater variation in gene copy number across species. Scatterplots displaying: **A** the relationship between the maximum gene number in each immune-related functional gene group (x-axis) and the range of copy number for each gene group across bee species (*n* = 70); and **B** the relationship between the maximum gene number in each immune-related functional gene group (x-axis) and the median absolute deviation (MAD) of copy number for each gene group across bee species (*n* = 70). Each dot represents an individual functional gene group. For each scatterplot, a best fit line and associated confidence intervals generated by a generalised additive model (GAM) are shown (Abbreviations for functional gene groups: AMP = Antimicrobial peptide; APHAG = Autophagy; ARK = Death-associated APAF1-related killer; CASP = Caspase; CAT = Catalase; CLIP = CLIP-domain serine protease; CTL = C-type lectin; FREP = Fibrinogen-like; GALE = Galectin; GNBP = Gram-negative binding protein/Beta-glucan recognition protein; IAP = IAP repeat, inhibitor of apoptosis domain; IGG = Immunoglobulin; IMD = Imd pathway; JAST = JAKSTAT; LYS = Lysozyme; ML = MD-2-related lipid recognition; NIM = NIMROD; PGRP = Peptidoglycan recognition protein; PPO = Prophenoloxidase; PRDX = Peroxidase; REL = Relish; SCR = Scavenger receptor; SOD = Superoxide dismutase; SPZ = Spaetzle; SRPN = Serine protease inhibitor; SRRP = Small RNA regulatory pathway; TEP = Thioester-containing protein; TOLL = Toll genes, Toll pathway)

Given that gene family expansion through duplication can drive evolutionary novelty and innovation [48], we examined the total number of genes within each immune-related gene group across bee species to determine groups with the greatest copy number variation. To assess the extent of copy number variation across families, we calculated both the range and the median absolute deviation (MAD) of gene counts. Together, these measures enable the identification of functional gene groups with large spreads in copy numbers across species (via increases in range) and broader variability across bee species (via increases in MAD). Accordingly, we calculated both metrics for gene counts within each immune-related gene group and investigated their relationship with overall gene group size (i.e., the maximum number of genes assigned to each group). This approach allowed us to test the hypothesis that larger immune-related functional gene groups may contain genes that experience divergent selective pressures due to functional or structural redundancy, potentially leading to increased structural divergence among members. Such divergence would be reflected in an increased range of gene counts across bee species. Additionally, if larger gene groups consistently exhibit greater variation in gene copy number across species, this would be reflected in higher MAD in copy number. As an initial analysis, we examined all orthogroups and found a strong association between orthogroup size and the range of gene numbers across species, partially supporting our hypothesis that larger immune-related orthogroups exhibit more extreme gene count variation (Generalised Additive Model [GAM]: edf = 8.96, F = 326,442, *p* < 2e-16; Additional file 2; Fig. S1A). A similarly significant relationship was found between orthogroup size and MAD of copy number (GAM: edf = 8.82, F = 861.4, *p* < 2e-16; Additional file 2: Fig. S1B). Within immune-related functional gene groups, we found that both the range (GAM: edf = 1, F = 31.46, *p* = 0.00000791; Fig. 2A), and MAD of copy number increase with gene group size (GAM: edf = 1.24, F = 9.35, *p* = 0.0019; Fig. 2B). In particular, across bee species, we found the greatest range in copy number for immune-related functional gene groups composed of functional categories containing multiple gene families, such as scavenger receptors (SCR: range = 11, min = 12, max = 23, median = 15 ± 1), and small RNA regulatory proteins (SRRP: range = 11, min = 26, max = 37, median = 29 ± 1), as well as for specific gene families, including CLIP-domain serine proteases (CLIP: range = 14, min = 15, max = 29, median = 20 ± 2), and serine protease inhibitors (SRPN: range = 11, min = 6, max = 17, median = 8 ± 1), respectively (Additional file 1: Table S2).

### Increased functional gene group size is largely associated with elevated structural diversity

**Fig. 3:**
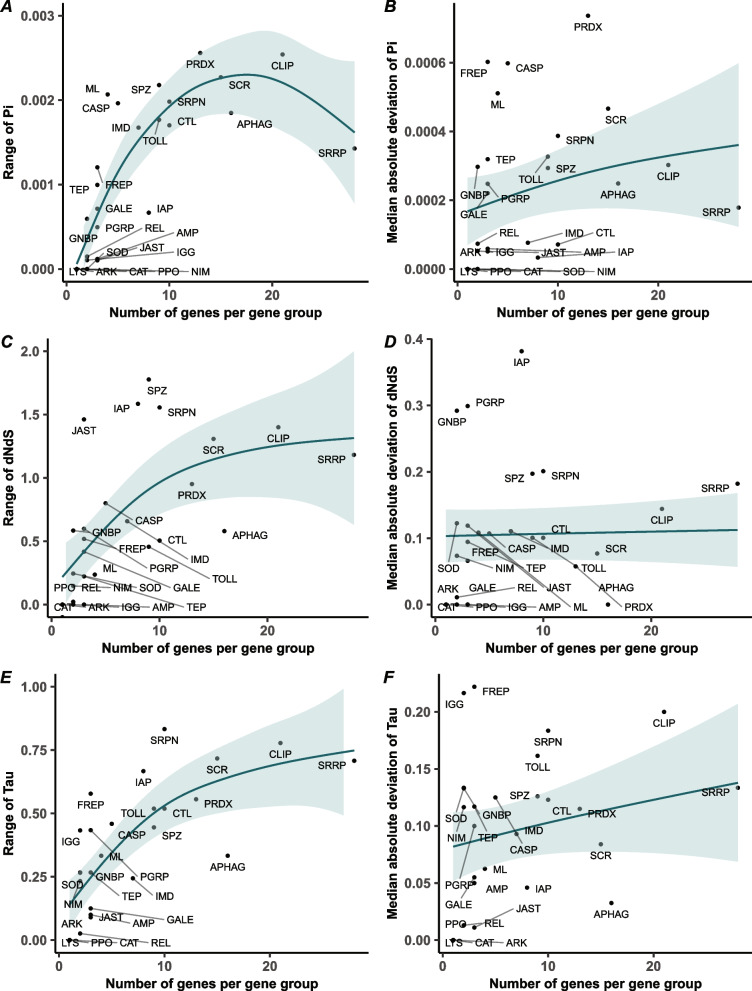
Nucleotide diversity, interspecific differentiation, and tissue-specificity scale non-linearly with immune-related functional gene group size in *Bombus terrestris*. Scatterplots displaying the relationship between: **A** the range of nucleotide diversity (measured in Pi) and the number of genes in each immune-related functional gene group; **B** the relationship between median absolute deviation (MAD) of nucleotide diversity [Pi] and the number of genes per gene group; **C** the range of interspecific differentiation (measured in dNdS, the ratio of nonsynonymous to synonymous substitutions between *B. terrestris* and the closely-related rusty patched bumblebee, *B. affinis*) and the number of genes per gene group; **D** the MAD of interspecific differentiation [dNdS] and the number of genes per gene group; **E** the range of tissue specificity (measured in tau) and the number of genes per gene group; and **F** the MAD of tissue specificity [tau] and the number of genes per gene group. Each dot represents an individual functional gene group while a best fit line and associated confidence intervals generated by a generalised additive model (GAM) are shown. (Abbreviations for functional gene groups: AMP = Antimicrobial peptide; APHAG = Autophagy; ARK = Death-associated APAF1-related killer; CASP = Caspase; CAT = Catalase; CLIP = CLIP-domain serine protease; CTL = C-type lectin; FREP = Fibrinogen-like; GALE = Galectin; GNBP = Gram-negative binding protein/Beta-glucan recognition protein; IAP = IAP repeat, inhibitor of apoptosis domain; IGG = Immunoglobulin; IMD = Imd pathway; JAST = JAKSTAT; LYS = Lysozyme; ML = MD-2-related lipid recognition; NIM = NIMROD; PGRP = Peptidoglycan recognition protein; PPO = Prophenoloxidase; PRDX = Peroxidase; REL = Relish; SCR = Scavenger receptor; SOD = Superoxide dismutase; SPZ = Spaetzle; SRPN = Serine protease inhibitor; SRRP = Small RNA regulatory pathway; TEP = Thioester-containing protein; TOLL = Toll genes, Toll pathway)

As immune genes evolve under different selective pressures [41, 49], gene duplication events contribute to copy number variation, potentially leading to not only variation in gene copy number but also in duplicated copies experiencing divergent selective pressures post-duplication. Therefore, it may be predicted that larger immune-related gene groups will exhibit elevated metrics of intraspecific genetic variation, as certain members potentially diverge under relaxed selection and accumulate higher numbers of mutations at the population level [50]. To assess this, we examined whether the size of an immune-related gene group correlates with differences in genetic variation among group members. Using publicly available population genomic data for the social bumblebee *B. terrestris* [51], we estimated nucleotide diversity (π) for each canonical immune gene (Additional file 1: Table S6) and examined the relationship between nucleotide diversity and gene group size. We found that the range of nucleotide diversity estimates generally increased with gene group size before dropping, a pattern which was driven by the SRRPs (GAM: edf = 8.2, F = 17.11, *p* = < 2e-16; Fig. 3A). Additionally, we found a significant, albeit weaker, relationship between the MAD of nucleotide diversity across gene group members and gene group size (GAM: edf = 8.2, F = 4.64, *p* = 0.0028; Fig. 3B).

As a complementary measure, we tested whether this pattern was also found in another bee species, the solitary, alfalfa leafcutting bee *Megachile rotundata* [52]. Similarly, both the range (GAM: edf = 2.5, F = 20.84, *p* = 1.32e-06) and MAD (GAM: edf = 1, F = 14.53, *p* = 0.00076) of nucleotide diversity in immune gene groups showed significant non-linear relationships with gene group size (Additional file 2: Fig. S2), suggestive of a possible conserved pattern across bee species.

As divergent selective pressures may also be reflected in interspecific changes in base composition, we examined the ratio of nonsynonymous to synonymous substitutions (dNdS, Additional file 1: Table S6) between *B. terrestris* and the closely-related rusty patched bumblebee, *Bombus affinis*. We found a significant non-linear relationship between the range of dNdS values and gene group size (GAM: edf = 2.39, F = 7.96, *p* = 0.00099), whereby values of dNdS per gene group increased before plateauing (Fig. 3C). However, we found no significant relationship between the MAD of dNdS values and gene group size (GAM: edf = 3.13, F = 1.34, *p* = 0.25; Fig. 3D), suggestive that while the range may differ with group size, the central variation in selective pressures across most members within a gene group does not systemically increase with group size. In addition, pairwise comparisons of dNdS ratios for representative species of other bee families, including the Megachilidae, Andrenidae, Halictidae, and Colletidae, found similar non-linear relationships between the range of dNdS values and gene group size (Additional file 1: Table S7; Additional file 2: Fig. S3), highlighting that interspecific differences in base composition increases with gene group size until a point where potential constraints occur.

As gene duplication can also lead to functional divergence, often reflected in differential gene expression among paralogues, we investigated whether immune-related gene groups of varying gene group size, also differ in tissue-specificity [53]. Using transcriptomic datasets from four *B. terrestris* tissues (brain, fat body, ovary, and other reproductive tissues, including spermatheca, oviduct, and vagina) [54], we calculated tau (Additional file 1: Table S6), a metric of tissue-specificity, for each putative canonical immune gene. Similar to patterns identified for intra- and interspecific genetic variation, we found a significant non-linear association between gene group size and tissue-specificity (GAM: edf = 2.56, F = 13.08, *p* = 2.43e-05; Fig. 3E), although variation in tissue-specificity within groups, measured in MAD, did not significantly change with group size (GAM: edf = 5.42, F = 1.63, *p* = 0.19; Fig. 3F).

**Fig. 4:**
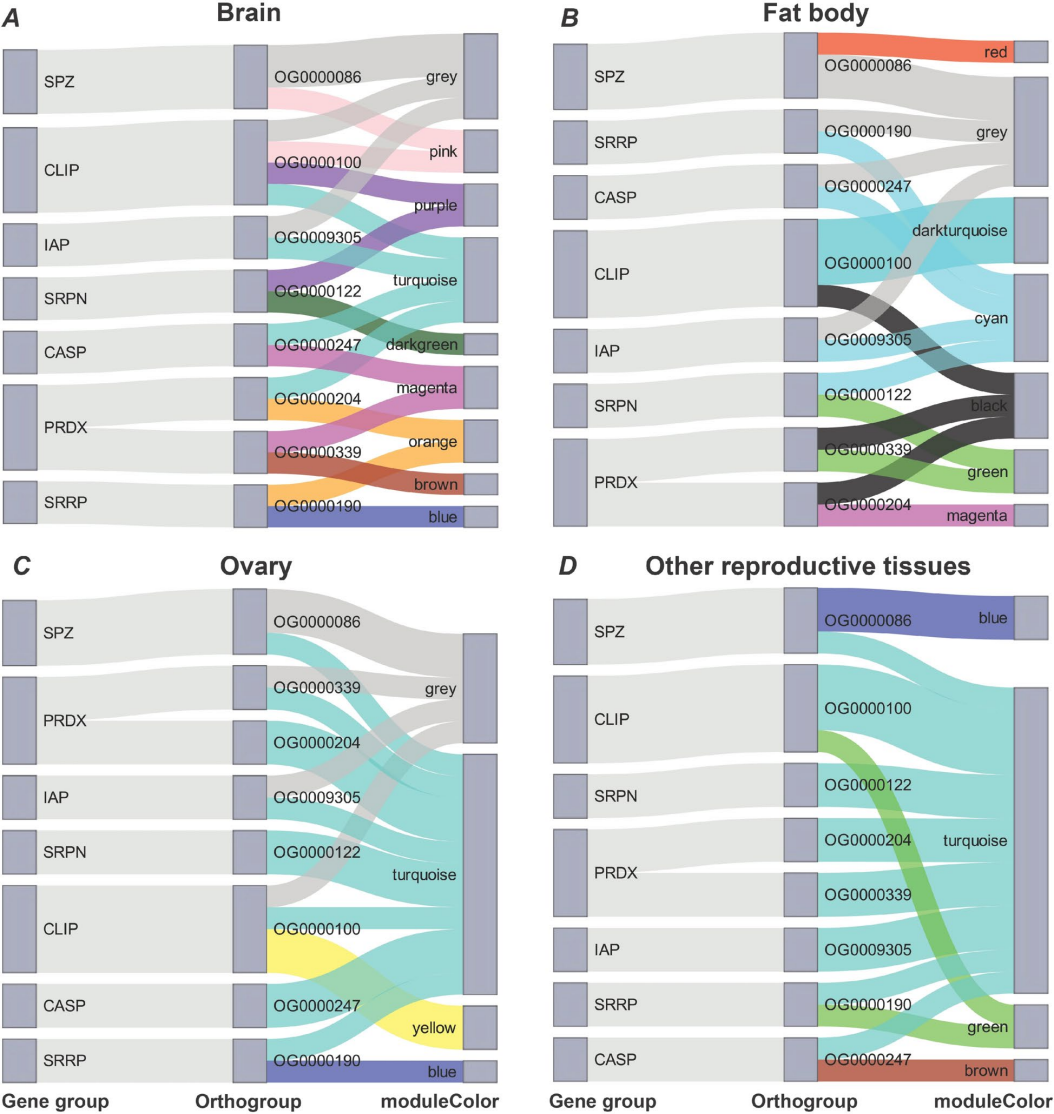
Assignment of immune-related paralogues to different co-expression modules suggests functional divergence within *Bombus terrestris*. Sankey plots showing that members of orthogroups (*n* = 8) that contained more than one immune paralogue from seven immune-related functional gene groups were classified into: **A** nine modules of brain genes; **B** seven modules of fat body; **C** four co-expression modules of ovary; and **D** four modules of genes of other reproductive tissues. (Abbreviations for gene groups: CASP = Caspase; CLIP = CLIP-domain serine protease; IAP = IAP repeat, inhibitor of apoptosis domain; PRDX = Peroxidase; SPZ = Spaetzle; SRPN = Serine protease inhibitor; SRRP = Small RNA regulatory pathway)

As a complementary approach, to further investigate whether immune-related gene group members display differential expression profiles, we constructed tissue-specific weighted co-expression networks using WGCNA for each of the four investigated tissues: brain (*n* = 11,502 genes used for construction of a weighted co-expression network), fat body (*n* = 11,370), ovary (*n* = 11,567), and other reproductive tissues (*n* = 11,284). The number of co-expression modules containing immune genes varied across tissues (brain = 18 modules; fat body = 10; ovary = 4; RTs = 7). For the purpose of identifying paralogues that demonstrate divergent expression profiles, we examined the assignment of canonical immune genes to orthogroups, finding the majority contained single immune genes (*n* = 152/160). However, for orthogroups containing multiple immune gene paralogues (*n* = 8/160), we observed divergent module membership (Fig. 4), indicating that despite close structural homology, paralogous immune genes are not consistently assigned to the same module. Specifically, all eight orthogroups with more than one immune paralogue showed split module membership in at least two tissues. Furthermore, paralogues from four of these orthogroups (50%) were assigned to different modules across all four tissues (Fig. 4), supporting post-duplication functional divergence.

### Lineage-specific losses of antimicrobial peptides across bees

**Fig. 5:**
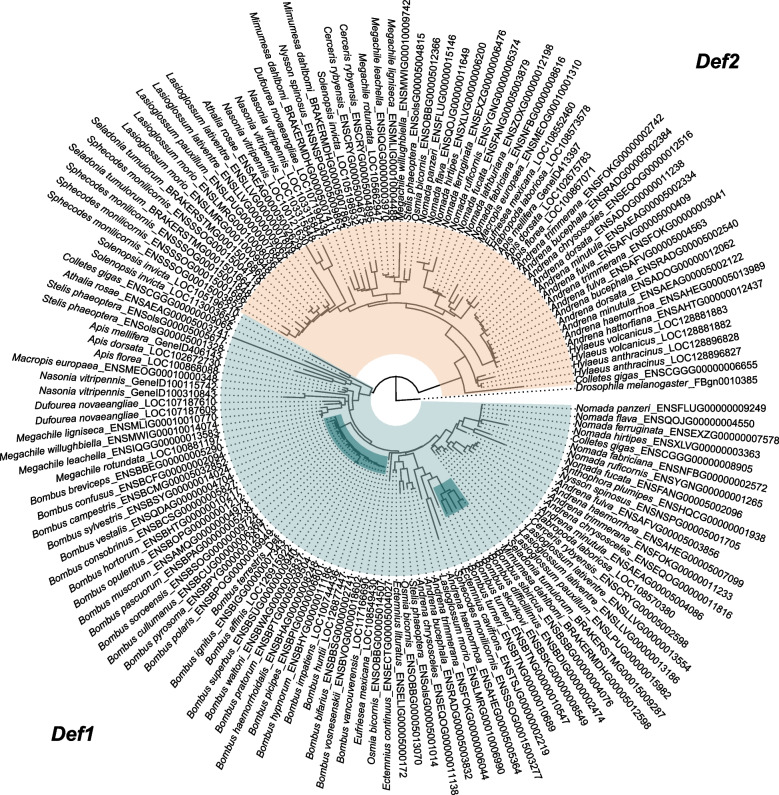
Phylogenetic relationship of *defensin* gene copies in bee species. A phylogenetic tree, generated based on identified homologues of *B. terrestris defensin*, displaying the evolutionary relationship with respect to conservation of two *defensin* gene copies (*Def1* (green) and *Def2* (tan)) in extant bee species. For each homologue, the species name, and Ensembl (Metazoa or Rapid) gene symbol are shown on the gene tree. Highlighted branches represented *Def1* homologues of *Bombus* species

While our homology-based analysis revealed broad conservation of immune gene groups across bee species (Fig. 1), we also identified evidence of gene loss events. Notably, we found that while two *defensin* copies, *Def1* and *Def2*, were generally conserved across bee species and other examined Hymenoptera, *Def2* was consistently absent in all 32 bumblebee species, which are representative of all 15 *Bombus* subgenera (Fig. 5; Additional file 1: Table S8; Additional file 2: Fig. S4). This expands on previous findings reporting the absence of *Def2* in the genome assemblies of the buff-tailed bumblebee (*B. terrestris*) and the common eastern bumblebee (*Bombus impatiens*) [37], highlighting that the loss of *Def2* likely occurred in the most recent common ancestor of all extant bumblebee species.

To further investigate the potential evolutionary consequences for the loss of *Def2* in bumblebees, we examined structural variation between the predicted proteins encoded by the two *defensin* copies in bees, which may underlie functional divergence [55]. Specifically, we identified conserved protein domains and motifs for both copies across all species in our analysis. Both genes encode for a conserved defensin domain (Defensin_invertebrate/fungal, IPR001542; Additional file 1: Table S9), while differences in motif architecture were identified whereby, we identified four motifs unique to *Def1*, with one motif specific to *Def2*, indicating structural differences between the two gene copies (Additional file 2: Fig. S5). Despite these differences, PAML-based analyses found no evidence of episodic positive selection acting on the ancestral branches of either copy (Likelihood Ratio Test: *p* > 0.09), suggesting that both genes have evolved under similar selective constraints prior to the loss of *Def2* in the bumblebees (Additional file 1: Table S10).

**Fig. 6:**
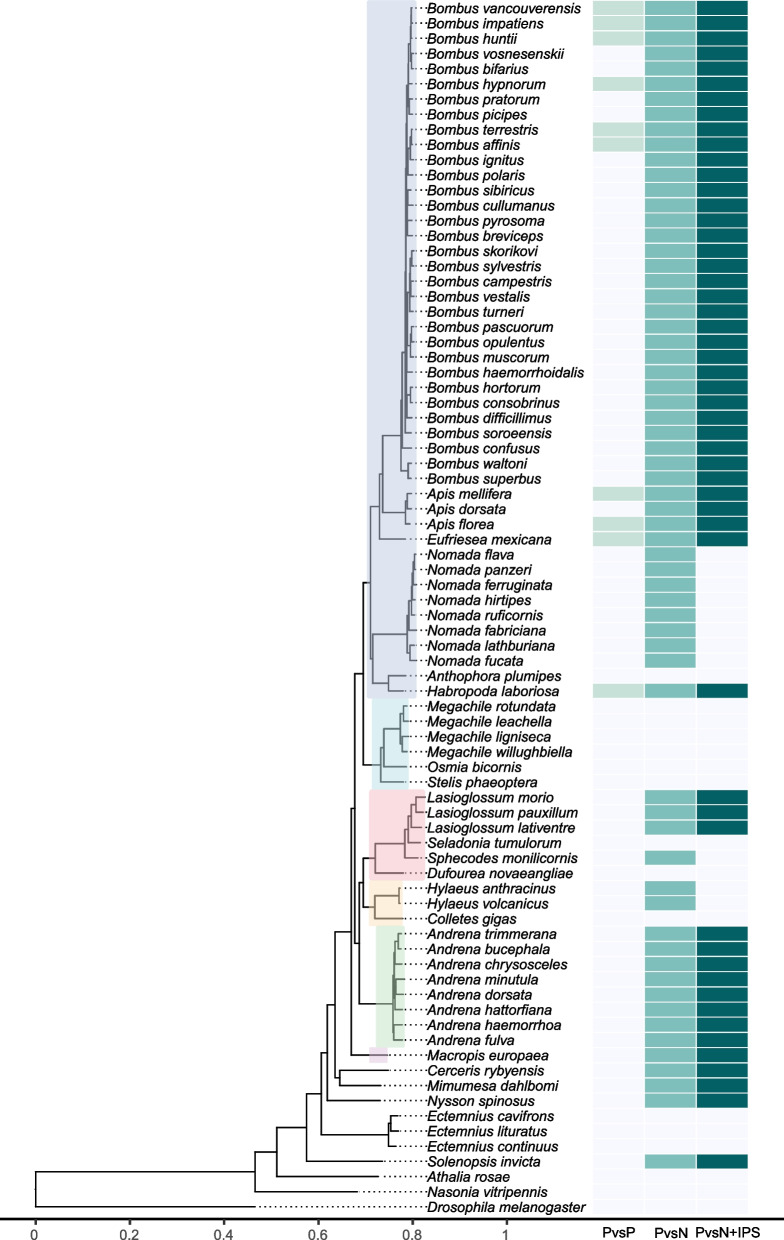
Putative independent lineage-specific losses of *abaecin* across bee species. A combined multi-panel plot displaying: the evolutionary relationships between investigated species are provided in a phylogenetic tree based on a homology-based comparative analysis of predicted proteomes for 80 species, including representatives of six bee families (Apidae = blue; Megachilidae = light blue; Halictidae = pink; Colletidae = light yellow; Andrenidae = light green; Melittidae = light purple); and a heatmap displaying the method and associated level of detection of putative *abaecin* copies. For investigation of conservation, we performed protein-to-protein homology-based analyses (“PvsP: OrthoFinder method), protein-to-predicted translated nucleotides (“PvsN”: tBLASTn method), and protein-to-predicted translated nucleotides followed by examination of predicted functional domains as annotated by InterProsScan (“PvsN + IPS”: a tBLASTn method, followed by targeted examination of the presence of the protein family domain Abaecin_antimicrobial_peptide, IPR012524). Individual colours correspond to individual levels of conservation at each level with grey indicating an absence of detection at each respective level

While the loss of one *defensin* gene appeared to be specific to bumblebees, our analysis also revealed putative lineage-specific losses of the antimicrobial peptide (AMP), *abaecin* (Fig. 6). Our initial homology-based analysis identified *abaecin* homologues in only 10 species. To refine this, we conducted protein-to-translated genome-based searches using tBLASTn, followed by domain-based investigation using InterProScan to detect conserved abaecin domains within genomic regions containing potential coding sequences. This approach identified credible abaecin-coding regions, defined by the presence of a predicted abaecin-associated protein domain (Abaecin_antimicrobial_peptide; IPR012524), which was predicted from the genomes of 42 more species (Fig. 6; Additional file 1: Tables S11 and 12). To further assess the presence of *abaecin* in these genome assemblies, we analysed RNA-seq data from ten of these 42 species, and found transcriptomic evidence supporting expression of *abaecin* based on alignment of reads to the predicted coding regions (Additional file 1: Table S13). In contrast, we found no evidence through these analyses for the presence of an *abaecin* homologue in the genome assemblies of all examined species from the Megachilidae (*n* = 6 species), Colletidae (*n* = 3), and within the genus *Nomada* of the Apidae (*n* = 8), suggesting independent loss events of this AMP across multiple bee lineages.

In comparison to *abaecin*, we found an inverse relationship with *hymenoptaecin*, where a homologue was identified in 95.7% of species (*n* = 67/70), which were collectively assigned to the same orthogroup, with 85.7% of species (*n* = 60) containing a single copy (Additional file 2: Fig. S6. Of the four described AMPs in bee species, putative *apidaecin* homologues were identified in two orthogroups, one that consisted of representatives from the honeybees (*n* = 3) and bumblebees (*n* = 30), while a second orthogroup, contained *apidaecin* homologues from members of five bee families (*n* = 28 species; Additional file 2: Fig. S7). All putative apidaecins were annotated with predicted functional domains for known apidaecins (Apidaecin, IPR004828).

## Discussion

Collectively, bees represent one of the most ecologically and commercially important insect groups through their provision of essential ecosystem services in the form of pollination. Crucial to their health is their immune system, which has evolved to help protect or reduce the impact of pathogens. Recent developments in genomic resources for bees, a group that consists of ~ 20,000 species [56], allow for comparative studies to understand the conservation in immune-related functional gene groups across extant bee families. Here, we performed genomic-based analyses incorporating seventy species, including representatives from six of the main seven bee families within the Anthophila, as well as representative species from closely related families in the larger Apoidea. Our analysis finds high levels of conservation of canonical immune gene groups across bee families with notable copy number variation in certain functional gene groups, including CLIP-domain serine proteases, serine protease inhibitors, and small RNA regulatory proteins. Using the bumblebee as a representative model, we find increases in the size of certain immune-related functional gene groups are associated with increases in intra- and interspecific variation, as well as differences in terms of tissue-specificity and extent of co-expression, yet the relationship is not fully linear indicating that there are constraints on the evolution of certain functional groups. Lastly, we expand upon previous findings on antimicrobial peptide (AMP) evolution in the bees, highlighting independent losses of important effector molecules within certain lineages.

Selective pressures imposed by pathogens on hosts can result in adaptive responses that enhance host fitness, primarily through increased tolerance or resistance [57]. A common mechanism for increasing functional diversity is gene duplication, often contributing to gene family expansions, including within immune-related gene families [58]. In our analysis, we identified variation in gene copy numbers in several immune-related functional groups across bee species, most notably the CLIP-domain serine proteases, serine protease inhibitors, and the antiviral-based small RNA regulatory protein (SRRP) gene group. These particular gene groups were not only the largest but also the most variable in terms of gene copy number across species. CLIP-domain serine proteases are enzymes that function in different facets of the insect immune response [59] and can be subcategorised into different groups (CLIPA-D), with each defined by the presence of one or more N-terminal CLIP domains [60]. These proteases are commonly present in insect haemolymph as inactive zymogens, which become activated in response to cellular damage [61] or pathogen presence [62]. In bees and other insects, certain serine proteases, such as easter, can trigger immune cascades, like the melanin-producing prophenoloxidase cascade [63], which plays key roles in the defence against macroparasites, as well as in wound healing [64]. In addition, CLIP-domain serine proteases may also contribute to the direct proteolysis of pathogens, activating components of the insect complement-like pathway [65], and supporting rapid responses. Coupled with their function are serpins, which were also found to vary in copy number across bee species, with expansions previously described in specific lineages, such as the bumblebees [37]. Serpins act by binding and inactivating serine proteases once a threat is removed, preventing self-damage or overreaction of immune responses [66, 67]. Beyond immune regulation, serpins can also assist in the activation of pro-spätzle, the ligand that binds and activates the Toll signalling pathway [17], which leads to the production of effector molecules, or is involved in the immediate or upstream activation of the proPO pathway. While a parsimonious explanation for the copy number variation of serpins observed across bee species may be to regulate expanded repertoires of serine proteases that function in the immune system, serpins also function in alternative roles, such as development [68], sperm storage [69], as well as roles in venom [70]. Potential specialisation or divergence in functional roles of serpin family members was supported through our own examination of gene expression profiles of serpins in *B. terrestris*, which provides evidence of certain members displaying higher tissue-specificity, a common fate post-duplication, as well as spatial variation in tissues, with possible roles in cognition (brain), reproduction (ovary, spermatheca), as well as immunity (fat body).

Compared to CLIP-domain serine proteases or serpins, SRRPs in our analyses of datasets for *B. terrestris* exhibited lower inter- and intraspecific variation with respect to range, which may reflect their cohesive functional role in small RNA pathways, including microRNAs, small-interfering RNA (siRNAs), and piwi-interacting RNA (piRNAs), which are involved in gene regulation [71], transposable element suppression [72], and antiviral defence [73]. While certain SRRPs may evolve under selective constraints, there is strong evidence from the literature to support positive selection acting on the micro- and macroevolutionary scales [37, 74, 75], which may contribute to the observed measures of intra- and interspecific diversity identified in our analysis. As bees are hosts to a number of debilitating viruses [76, 77], SRRPs may evolve under strong positive selection to aid antiviral defence [37]. In addition, gene duplications of SRRPs have been described in other insect species [78], providing a potential basis for functional innovation. Therefore, duplication events may also contribute to the variation in gene copy number observed across bee species in our analysis. However, our analysis suggests that structural variation in SRRPs is more constrained compared to the immune-related gene groups that are composed of families, which may experience higher levels of turnover [79].

While our analysis highlighted copy number variation, which may be attributed to gene duplication events, gene loss is also an important evolutionary process. Such losses can occur when environmental or lifestyle changes reduce the selective pressure acting on specific genes [80]. Although pseudogenisation and gene loss are frequent fates of gene duplicates [81], we also found apparent cases of gene loss wholly or partially independent of duplication events, particularly in AMPs, such as *abaecin* and *defensin*. Losses of *defensin* genes have been previously reported in bumblebees [37], which our findings support and extend by suggesting that gene loss likely arose in the common ancestor of extant bumblebee species (Fig. 5)*.* In contrast, the closely related honeybee *A. mellifera* retains two *defensin* copies, which appear to have diverged functionally, as reflected in structural differences between predicted proteins [56]. This divergence is consistent with an ancestral gene duplication event, with subsequent gene loss likely in bumblebees due to relaxed selective pressures. For example, one of the *A. mellifera* defensins, also known as royalysin, is secreted into honey and royal jelly, both of which are absent from bumblebee biology [82]. This functional difference may point towards variation in social structure and life-history traits that could contribute to gene loss, as predicted to occur under social immunity-related hypotheses where components of the immune system evolve under relaxed selection as a consequence of functional redundancy due to the action of collective colony-level hygienic behaviour [35]. However, the presence of more than one *defensin* copy in other non-social bees (Fig. 5) indicates alternative functions beyond the activities for which they are used by honeybees. While many solitary bees provision their developing young with food resources, it may be the case that such bees secrete antimicrobial peptides to aid in initial nest sterilisation prior to egg laying or represent products to sterilise collected resources [83]. However, again, as bumblebees also perform active brood care as akin to what is displayed in honeybees, it likely removes this hypothesis as an argument for loss. Losses may also occur due to functional redundancy, where one AMP performs a function analogous to another, loss or escape of a pathogen, or a change in microbial community coinciding with immunological changes. In terms of their mode of action, defensins are cationic peptides that bind to the surface of positively charged microbial membranes, such as those of Gram-positive bacteria [84]. They bind to the surface of a microbial target, integrate into the membrane resulting in pore formation and destabilisation, followed by cell lysis, and eventual death. Bees also possess another AMP with a described role in active microbial membrane disruption, which is hymenoptaecin. Like defensin, hymenoptaecin is inducible in response to infection, displaying broad spectrum activity against both Gram-positive and -negative bacteria [85]. Studies examining the activity of such genes at the transcript and protein level have highlighted that hymenoptaecin and defensin are both increased in response to immune challenge [86, 87]. Therefore, they may either act synergistically to remove pathogens or one copy may compensate for the loss of the other in other species.

Another protein that hymenoptaecin does interact with is abaecin, a proline-rich AMP with described properties against Gram-negative bacteria [84, 88]. In comparison to the membrane disruptive properties of defensin and hymenoptaecin [85], abaecin has been shown to disrupt bacterial translation leading to cell death [89]. It is reliant on other AMPs, such as hymenoptaecin, to create pores in bacterial membranes so it can enter and interact with the ribosomal machinery of the cell [90]. An initial loss of *abaecin* was first highlighted in the red mason bee, *Osmia bicornis* [39], and more recently in *Megachile rotundata* [91], while, here, our analyses expand this to other members of the Megachilidae, indicating that the gene was likely lost in a common ancestor. Furthermore, our analysis highlighted independent losses of *abaecin* within members of other families, such as the Colletidae and Apidae, most notably among extant members of the *Nomada*, indicating that the gene may have been independently lost at different times across different bee families. The genus *Nomada* consists of a group of kleptoparasites that parasitise ground-nesting hosts of the Andrenidae [92]. Reduction in immune genes may occur within social parasites as their interaction with the environment is reduced compared to hosts as they do not collect pollen, and, therefore, avoid sites, such as flowers, where transmission of pathogens may occur [93]. This could lead to relaxed selection acting on certain immune genes. However, if this was the case, we may have found potentially similar patterns in other analysed bee species that display parasitic life-cycles (e.g., cuckoo bumblebees), which was not the case. Similar levels of AMPs were reported in previous studies of the Megachilidae, such as *M. rotundata* [37, 94], yet here, as previously reported for *O. bicornis* [39], we find no evidence either within the predicted proteome or underlying genomic sequences meaning that gene loss is likely evolutionarily old as we do not find evidence of remnants. Similarly, the absence of a putative *abaecin* homologue in multiple genome assemblies from members of the Megachilidae suggests that the missing gene is not the consequence of an assembly artefact or error in gene model prediction. Loss of certain AMPs may be compensated by other components of the immune system or the presence of other antimicrobial peptides that are either undescribed or taxonomically restricted, such as AMPs that have been described in the venom of members of the Megachilidae [83], Apidae [95–98], and Colletidae [99], which possess antimicrobial activity. However, it is yet to be determined if such genes play an active role in the immune system of bees, although roles in social immunity have been proposed [100]. Future functional research should investigate whether these venom-associated peptides contribute to immunity, potentially compensating for the loss of canonical AMPs like *abaecin*.

The loss of AMPs, particularly in species that contain low numbers of AMP-encoding genes, raises the question of why lose these genes? Gene loss may occur through neutral processes, such as in regressive evolution, where loss of function of genes coding for defunct traits occurs gradually over time [101]. Alternatively, gene loss may be adaptive to remove redundant or dispensable genes from the genome, such as the ‘less-is-more-hypothesis’ [102], which is proposed to increase energetic savings, as well as spatial efficiency. Gene loss within the immune system of other species (e.g., vertebrates [103]) has been attributed to environmental shifts, such as if a pathogen that exerts a strong selective pressure is removed, which may have occurred in certain bee lineages resulting in losses. For bees, their haplodiploid nature is also predicted to contribute to rapid loss of deleterious or even redundant alleles [104], yet population genomic screens have highlighted that potential loss-of-function alleles are not immediately removed [41], which may also explain why we find remnants of certain AMPs in the genomes of these lineages. Regardless of the evolutionary mechanism, the consequences, if any, of gene loss of antimicrobial peptides requires further investigation.

Our research focuses on the variation of immune-related gene groups across multiple bee families, with each immune gene identified through a homology-based analysis. However, the quality of genome assemblies and annotations can influence gene identification, which we have attempted to account for through comparisons of NCBI and Ensembl datasets (Additional file 1: Table S14) yet can still pose a problem. This is a particular issue for non-model organisms where RNA-seq and proteomic datasets are limited or absent for gene model predictions meaning that certain genes may be missed. Similarly, orthogroup assignment may also lead to genes being misclassified, or unassigned in our analysis. In terms of determining variation in copy number across gene groups, we calculated both the range and median absolute deviation. While MAD is generally a more robust measure of variation compared to the range, it can be susceptible to small sample sizes. As many immune gene groups consist of four or less genes, this can inflate noise in MAD estimates. An additional complication is certain biases in terms of representatives in our dataset with many bees deriving from the Apidae, which although is the largest bee family, may underestimate certain patterns found in other bee families. Lastly, a number of our analyses were restricted to certain species, such as our tissue-specific expression analyses, meaning further research is required to determine if patterns reported here are general patterns associated with bees or species-specific characteristics.

## Conclusions

Given the concerns over the impact known and emerging pathogens can have on pollinator health [28, 105], improving our understanding of the evolution and expression of the canonical immune gene groups of bees is of timely importance. Taking advantage of the increasing availability of genome assemblies for representative species across the bee phylogeny, we demonstrate high levels of conservation of key canonical immune gene groups across members of extant bee families, as well as highlight heightened copy number variation in specific functional gene groups, including lineage-specific losses. With increasing genomic resources, platforms are being generated to improve our understanding beyond traditional study systems of bee biology. Similarly, while our analysis focussed on canonical immune gene groups, increases in transcriptomic and proteomic studies examining functional expression of the immune genes in more bee species will provide the basis for exploring and determining non-canonical immune genes across the bees as a group. Such resources will also provide the means to perform comprehensive analyses to determine how pathogens, abiotic environmental conditions, as well as major evolutionary transitions (e.g., eusociality) have shaped the evolution of the immune system in bees. Ultimately, improving our understanding of bee immunity, as well as their pathogens, is required to fundamentally determine their capacity and resilience to respond to ongoing and increasing environmental threats, which is crucial to ensure the maintenance of vital ecosystem services.

## Methods

### Identification of putative canonical immune genes across bee species

To investigate the conservation of the genetic bases of the innate immune system of bee species, we used a homology-based approach performed through OrthoFinder v2.5.5 [106] to identify putative canonical immune genes in 70 bee species from six families: Andrenidae (*n* = 8 species), Apidae (*n* = 46), Colletidae (*n* = 3), Halictidae (*n* = 6), Megachilidae (*n* = 6), and Melittidae (*n* = 1); Additional file 1: Table S1). Furthermore, to determine possible immune genes, we selected *B. terrestris* as our reference species, as canonical immune genes have been described for this species, are publicly available, and the identifiers (i.e., gene symbols) still largely exist [37, 38]. While putative canonical immune genes were described earlier for the western honeybee *A. mellifera* [107], the gene symbols are no longer in use meaning it is difficult to directly track annotations in later genome assemblies [108]. Since a second reference genome assembly for *B. terrestris* (iyBomTerr1.2) was generated in 2022 and made public through the Ensembl Metazoa database [109], we determined each canonical immune gene, as inferred in the original genome assembly, in the reference predicted proteome of the new reference genome assembly by using BLASTp (e-value threshold: 1e-06) [110] based on best assigned bit-score. As part of our analysis, we also included six digger wasps from the superfamily Apoidea, as well as a wasp (*Nasonia vitripennis*), an ant (*Solenopsis invicta*), a sawfly (*Athalia rosae*), and also the fruit fly, *Drosophila melanogaster*, to serve as outgroups in our OrthoFinder-based analysis. For our analysis, we obtained predicted protein sequences from the Ensembl Rapid Release database [111] with the exception of *B. terrestris*, *A. mellifera*, *N. vitripennis*, *S. invicta*, and *A. rosae,* which were obtained from the Ensembl Metazoa database [112]. The full list of species used, as well as respective assembly name, accession ID, and summary statistics for assembly metrics, are provided in Table S1 of Additional file 1.

To perform our homology-based analysis, for each species, we extracted the longest protein isoform per gene using the script ‘primary_transcript.py’ provided by OrthoFinder. Using these subsetted files, we ran OrthoFinder using all predicted genes for our species of interest with the ‘-M msa’ parameter for species tree inference, which is computationally more intensive but generates more accurate results. For the other steps, we used the default parameters, based on author recommendations.

As part of its analysis, OrthoFinder produces orthogroups, which consists of groups of putative homologous genes. Therefore, if an orthogroup contains an annotated canonical immune gene from *B. terrestris*, we extracted putative immune gene homologues in each species. To increase confidence of these putative homologues, we also annotated predicted proteins of each orthogroups using InterProScan [46] as it would be predicted that homologues would carry conserved functional domains. Lastly, as gene model predictions can influence comparative analyses [113], we also performed a complementary OrthoFinder-based analysis using 23 species, which also had predicted proteins from the NCBI Eukaryotic Genome Annotation pipeline, which are housed on the NCBI Reference Sequence (RefSeq) database. We found an identical number of putative immune genes in 15 out of 23 species (Additional file 1: Table S14) with no significant overall difference in gene counts between annotation sources (Generalised Linear Model: *p* > 0.9), suggestive of our analyses being robust to choice of annotation source, as gene counts were largely consistent across datasets. However, caution is warranted for certain species where notable differences in gene annotations were observed, underscoring the importance of careful annotation selection in comparative genomic studies (Additional file 2: Fig. S8).

### Investigation and verification of genes missed by OrthoFinder

Since the majority of gene model predictions of recent genome assemblies are computationally inferred, they may contain fragmented or missing gene models. As a result, predicted proteins may be truncated or absent due to incorrect identification of start or stop codons. To further assess whether genes absent in our homology-based analysis were still present in the genome assemblies of candidate species, we used tBLASTn [110] to search the predicted proteins of *B. terrestris* against the predicted translated genome sequences of each species. For matched regions, we extracted the nucleotide sequences, generated their predicted amino acid translations, and annotated them using InterProScan to determine if putative coding regions coded for functional domains.

### Intraspecific variation across immune-related functional gene groups

As immune genes and associated groups may also experience divergent selective pressures at the population level, resulting in microevolutionary signatures identifiable at the genomic level, we compared measures of nucleotide diversity across immune-related functional gene groups for two bee species from different bee families: the bumblebee, *B. terrestris* (Apidae), and the alfalfa leafcutting bee, *M. rotundata* (Megachilidae). In brief, the bumblebee data were generated by Kardum Hjort et al. (BioProject PRJEB49221) [51], which consisted of 95 workers sampled from either the natural environment or commercially imported into Sweden. For *M. rotundata*, the data were generated by Jones et al. (BioProject PRJNA508875) [52]. The dataset consisted of 25 samples sampled from three American locations. For both species, we obtained publicly available raw FASTQ files from the NCBI Sequence Read Archive using the SRA-Toolkit (v.2.10.9) [114]. Next, we filtered raw reads using fastp (v.0.20.1) [115] using default parameters. We then aligned filtered reads against their respective genome assemblies using bwa-mem (v.0.7.17) [116]. The resulting alignment (BAM) files were sorted using samtools and putative optical duplicates identified and marked using Picard’s MarkDuplicates function (v.2.6.0). After deduplication, genome-wide coverage and the number of aligned reads were calculated using Bedtools (v.2.29.2) [117]. Using all aligned BAM files, we performed variant calling using freebayes-parallel (v.1.3.6) [118] using default parameters. Variant filtering was subsequently performed for each species using VCFtools (v.0.1.16; parameters = –max-alleles 2, –min-alleles 2, –max-missing 1, –remove-indels, –maf 0.05) [119], which retained biallelic single nucleotide polymorphisms present in all samples. Using a sliding windows-based approach, for each species, we calculated nucleotide diversity (pi) estimates within 10 kb windows across the genome using VCFtools. To generate nucleotide estimates for canonical immune genes, we intersected windows that overlapped with genomic coordinates of putative immune genes and calculated the mean measure of pi across overlapping windows as a representative measure for a gene.

### Interspecific variation across immune-related functional gene groups

To investigate the evolutionary divergence of putative immune genes across multiple bee families, we estimated the ratio of nonsynonymous (dN) to synonymous (dS) substitutions (dNdS) between two closely related species within each family. Specifically, we compared coding sequences of *B. terrestris* and *B. affinis* (Apidae), *Megachile leachella* and *Megachile rotundata* (Megachilidae), *Andrena fulva* and *Andrena haemorrhoa* (Andrenidae), *Lasioglossum pauxillum* and *Lasioglossum morio* (Halictidae), and *Hylaeus volcanicus* and *Hylaeus anthracinus* (Colletidae) [120]. For these pairwise comparisons, we used the coding sequence for the longest predicted protein isoform as identified and used by OrthoFinder, and calculated dNdS ratios for protein-coding genes using the ‘dNdS()’ function from the R package orthologr v.0.4.2 [121] with default parameters. As part of the orthologr-based analysis, putative orthologous sequences between each species pair were first identified using the reciprocal best hit (RBH) method implemented in DIAMOND2 [122] (e-value threshold of 1E-5), with the ‘Comeron’ method [123] used to estimate dNdS values.

### Tissue-specificity-based analyses immune-related functional gene groups

To understand whether immune-related functional gene groups differ in their range of expression patterns across different tissues of putative immune groups, which may reflect localisation, we used available datasets generated by Zhuang et al. [54]. An entire breakdown of sample numbers and tissues is provided in Table S15 of Additional file 1. As calculated in a previous study [43], we extracted tau values (representing tissue-specificity) of each canonical immune gene, which was estimated using normalised expression values across four tissues (brain, fat body, ovary, and other reproductive tissues) sampled for *B. terrestris*.

### Comparison of gene group size and measures of structural and functional divergence

To assess putative signatures of structural and functional divergence, we calculated both the range, and median absolute deviation (MAD) for pi (nucleotide diversity), dNdS (interspecific divergence), and tau (tissue-specificity) for members of each immune-related functional gene group. We then assessed the relationship with the maximum number of genes per functional gene group, as it can be predicted that larger gene groups may allow for greater structural divergence within a species (i.e., higher pi) and between species (i.e., higher dNdS), as well as functional divergence in gene expression across tissues, which would be reflected in larger ranges of tissue-specificity. In addition, larger variation in expression profiles that would be equivalent amongst gene group members, would also be reflected in greater measures of MAD. To determine the relationship of gene group size and each of these metrics, we performed individual generalised additive models (GAMs) implemented in the R package mgcv (v. 1.9–1) [124]. As a complementary measure, we also performed individual linear models (LMs) using the R package mgcv, with the results of the LMs available in Additional file 1: Table S16.

### Weighted gene co-expression network analysis of canonical immune genes

As transcriptional divergence among gene group members may also be reflected in assignment to different modules of a co-expression network, we examined expression profiles across immune-related functional gene groups by reanalysing results from weighted gene co-expression network analyses (WGCNA) that we previously performed independently for four tissues (brain, fat body, ovary, and other reproductive tissues, including spermatheca, oviduct, and vagina) of *B. terrestris* [43]. Within each tissue, we first transformed gene-level counts using variance stabilizing transformation (VST) provided by the R package DESeq2 (v.3.16) [125]. We then performed sample clustering for the count data using the base R function ‘hclust()’ and removed any identified outliers. We generated an adjacency matrix using the soft threshold power (β), determined with the ‘pickSoftThreshold’ and ‘scaleFreePlot’ functions from the WGCNA package (v.1.72–5) [126]. This adjacency matrix was then transformed into a topological overlap matrix (TOM), which was further converted into a topological dissimilarity matrix (1-TOM). Genes were subsequently clustered into different eigengene modules (minClusterSize = 30; deepSplit = 2) using the ‘hclust()’ function. Highly correlated modules were merged using a cut height of 0.25. Finally, we identified the modules in which canonical immune genes clustered, allowing us to examine their expression profiles across orthogroups and gene families.

### Assessment of structural divergence and loss of antimicrobial peptides

To preliminarily assess why certain genes, such as antimicrobial peptides, were lost in specific lineages, we further investigated divergence both between putative paralogues, and across species based on comparison of conserved functional domains and predicted protein motifs. For functional domains, we examined the presence and conservation of predicted functional domains associated with *defensin* (Defensin_invertebrate/fungal, IPR001542), and *abaecin* (Abaecin_antimicrobial_peptide; IPR012524). In addition, for the two *defensin* copies found across bee species, we assessed motifs of predicted protein sequence of *defensin* using the MEME Suite 5.5.8 [127]. We then visualised probable motifs using TBtools [128] with phylogenetic relationships guided by each *defensin* gene tree inferred by OrthoFinder.

### Investigation of positive selection acting on defensin within Hymenoptera

To assess signatures of episodic positive selection acting on either copy of *defensin*, we used the CODEML module of PAML v.4.10.7 [129]. For this analysis, we used a rooted *defensin* gene tree*,* generated by OrthoFinder. We next kept only the topology of the gene tree and transformed it to an unrooted phylogenetic tree using the R package ape v.5.8 [130]. Predicted coding sequences of *defensin* homologues from all species were next aligned using PRANK v.170427 [131], and the resulting alignment was pruned using Gblocks v.0.91b [132]. We then applied branch-site models to test for evidence of positive selection on the ancestral branch of the two copies of *defensin*. Specifically, we compared an alternative model (model = 2, NSsites = 2, fix_omega = 0, omega = 0.5) with a null model (model = 2, NSsites = 2, fix_omega = 1, omega = 1) to assess whether either gene copy shows evidence of positive selection across bee lineages, following parameters recommended by the authors of PAML [133].

## Supplementary Information

Additional file 1.

Additional file 2.

## Data Availability

All the genome assemblies used for the comparative genomic analyses are publicly available in the Ensembl Rapid Release database (https://rapid.ensembl.org/index.html), the Ensembl Metazoa database (https://metazoa.ensembl.org/index.html), and the National Center for Biotechnology Information (NCBI) GenBank database, with the full list of genome assemblies provided in the supplementary information (Additional file 1). The RNA-seq data of *B. terrestris* used in the present study are publicly available from the NCBI Sequence Read Archive (SRA) database (BioProject Accession: PRJNA868857 [134]). The population genomic data were also obtained from the NCBI SRA database (*Bombus terrestris*: BioProject Accession: PRJEB49221 [135], *Megachile rotundata*: PRJNA508875 [136]).

## Abbreviations

dNdS: The ratio of nonsynonymous to synonymous substitutions
GAM: Generalised additive model
GLM: Generalised linear model
LM: Linear model
MAD: Median absolute deviation
pi: Nucleotide diversity
TOM: Topological overlap matrix
VST: Variance stabilizing transformation
WGCNA: Weighted gene co-expression network analyses

## Canonical immune-related functional gene group

AMP: Antimicrobial peptide
APHAG: Autophagy
ARK: Death-associated APAF1-related killer
CASP: Caspase
CAT: Catalase
CLIP: CLIP-domain serine protease
CTL: C-type lectin
FREP: Fibrinogen-like
GALE: Galectin
GNBP: Gram-negative binding protein/Beta-glucan recognition protein
IAP: IAP repeat, inhibitor of apoptosis domain
IGG: Immunoglobulin
IMD: Imd pathway
JAST: JAKSTAT
LYS: Lysozyme
ML: MD-2-related lipid recognition
NIM: NIMROD
PGRP: Peptidoglycan recognition protein
PPO: Prophenoloxidase
PRDX: Peroxidase
REL: Relish
SCR: Scavenger receptor
SOD: Superoxide dismutase
SPZ: Spaetzle
SRPN: Serine protease inhibitor
SRRP: Small RNA regulatory pathway
TEP: Thioester-containing protein
TOLL: Toll genes, Toll pathway
