# Supplementary material for: The evolution of the immune system of bees is defined by conservation, expansions, and losses
Source: BMC Biol. 2026 Mar 10;24:94. doi: 10.1186/s12915-026-02564-0 (PMC13063555; doi:10.1186/s12915-026-02564-0)
Supplement: Supplementary file 2 — Additional file 2. [file 12915_2026_2564_MOESM2_ESM.pdf]

The evolution of the immune system of bees is defined by conservation, expansions, and losses

Hongfei Xu[1]*, Ina Köhler[1] & Thomas J. Colgan[1,2]*

1. Institute of Organismic and Molecular Evolution, Johannes Gutenberg University Mainz, Hanns-Dieter-Hüsch-Weg 15, 55128 Mainz, Germany.
2. Institute of Quantitative and Computational Biosciences (IQCB), Johannes Gutenberg University Mainz, Hanns-Dieter-Hüsch-Weg 15, 55128 Mainz, Germany.

Email address:

Hongfei Xu: honxu@uni-mainz.de

Ina Köhler: inkoehle@uni-mainz.de

Thomas J. Colgan: tcolgan@uni-mainz.de

Corresponding authors: Hongfei Xu and Thomas J. Colgan

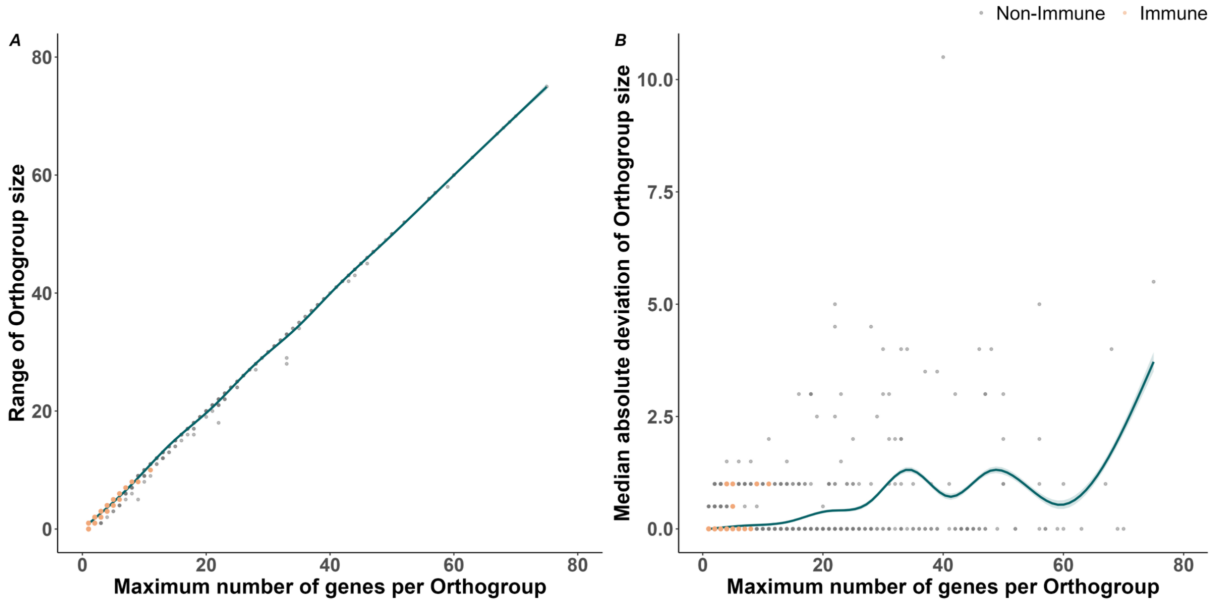

Fig. S1. **Larger orthogroups show greater variation in gene copy number across species.** Scatterplots displaying: **A)** the relationship between range and the maximum value of orthogroup size across all species; **B)** the non-linear relationship between median absolute deviation and the maximum value of orthogroup size across all species. The orthogroups containing immune genes are highlighted in tan. For each scatterplot, a best fit line and associated confidence intervals generated by a generalised additive model (GAM) are shown.

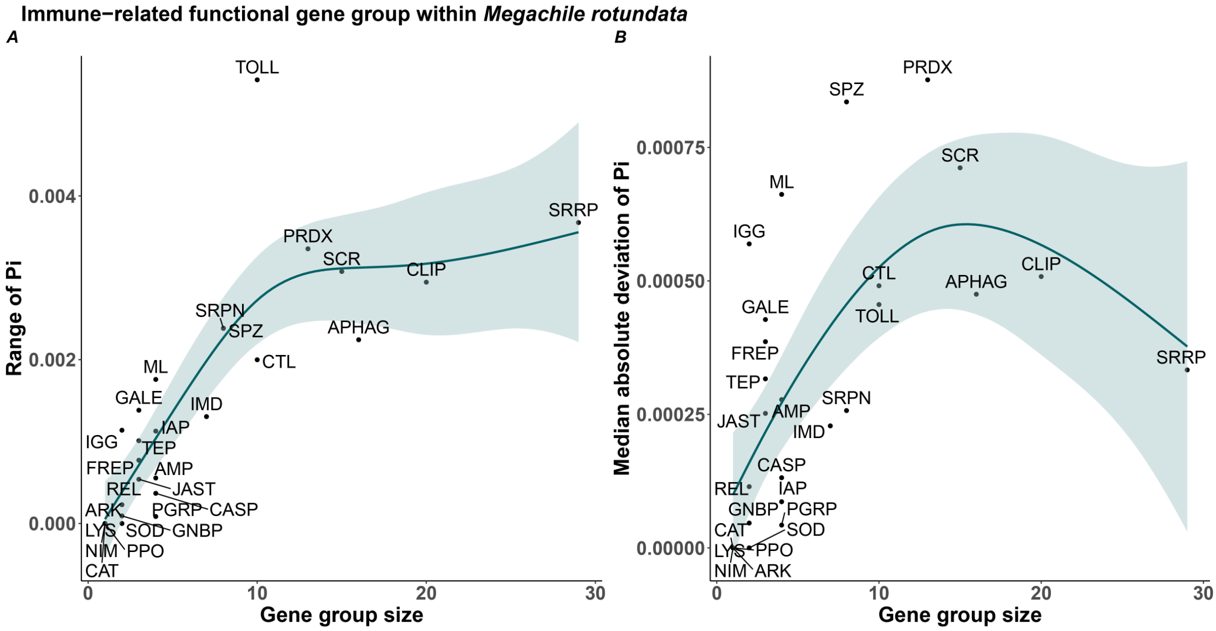

Fig. S2. **Nucleotide diversity scales non-linearly with immune-related functional gene group size in *Megachile rotundata*.** Scatterplots displaying: **A)** the non-linear relationship between range of genetic diversity (measured in Pi) and the number of genes in each immune-related functional gene group; **B)** the non-linear relationship between MAD of genetic diversity (measured in Pi) and the number of genes in each immune-related functional gene group. For each scatterplot, a best fit line and associated confidence intervals generated by a generalised additive model (GAM) are shown (Abbreviations: AMP = Antimicrobial peptide; APHAG = Autophagy; ARK = Death-associated APAF1-related killer; CASP = Caspase; CAT = Catalase; CLIP = CLIP-domain serine protease; CTL = C-type lectin; FREP = Fibrinogen-like; GALE = Galectin; GNBP = Gram-negative binding protein/Beta-glucan recognition protein; IAP = IAP repeat, inhibitor of apoptosis domain; IGG = Immunoglobulin; IMD = Imd pathway; JAST = JAKSTAT; LYS = Lysozyme; ML = MD-2-related lipid recognition; NIM = NIMROD; PGRP = Peptidoglycan recognition protein; PPO = Prophenoloxidase; PRDX = Peroxidase; REL = Relish; SCR = Scavenger receptor; SOD = Superoxide dismutase; SPZ = Spaetzle; SRPN = Serine protease inhibitor; SRRP = Small RNA regulatory pathway; TEP = Thioester-containing protein; TOLL = Toll genes, Toll pathway).

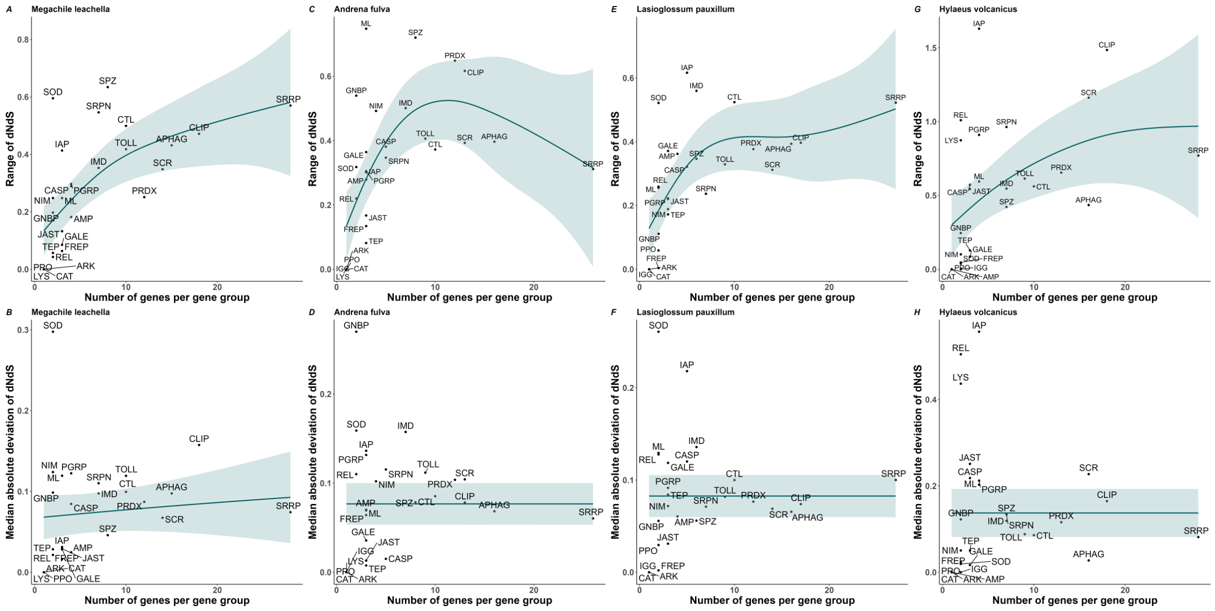

Fig. S3. **Interspecific differentiation scales non-linearly with immune-related functional gene group size in several bee families**. **A)** a non-linear relationship between the range of interspecific differentiation (measured in dNdS, the ratio of nonsynonymous to synonymous substitutions between *Megachile leachella* and the closely-related *Megachile rotundata*.) and the number of genes in each immune-related functional gene group; **B)** the non-linear relationship between Median absolute deviation (MAD) of interspecific differentiation (measured in dNdS) and the number of genes in each immune-related functional gene group; **C)** a non-linear relationship between the range of interspecific differentiation (measured in dNdS, the ratio of nonsynonymous to synonymous substitutions between *Andrena fulva* and the closely-related *Andrena haemorrhoa*.) and the number of genes in each immune-related functional gene group; **D)** the non-linear relationship between MAD of interspecific differentiation (measured in dNdS) and the number of genes in each immune-related functional gene group; **E)** a non-linear relationship between the range of interspecific differentiation (measured in dNdS, the ratio of nonsynonymous to synonymous substitutions between *Lasioglossum pauxillum* and the closely-related *Lasioglossum morio*) and the number of genes in each immune-related functional gene group; **F)** the non-linear relationship between MAD of interspecific differentiation (measured in dNdS) and the number of genes in each immune-related functional gene group; **G)** a non-linear relationship between the range of interspecific differentiation (measured in dNdS, the ratio of nonsynonymous to synonymous substitutions between *Hylaeus volcanicus* and the closely-related *Hylaeus anthracinus*) and the number of genes in each immune-related functional gene group; **H)** the non-linear relationship between MAD of interspecific differentiation (measured in dNdS) and the number of genes in each immune-related functional gene group; Each dot represents an individual functional gene group. For each scatterplot, a best fit line and associated confidence intervals generated by a generalised additive model (GAM) are shown (Abbreviations for functional gene groups: AMP = Antimicrobial peptide; APHAG = Autophagy; ARK = Death-associated APAF1-related killer; CASP = Caspase; CAT = Catalase; CLIP = CLIP-domain serine protease; CTL = C-type lectin; FREP = Fibrinogen-like; GALE = Galectin; GNBP = Gram-negative binding protein/Beta-glucan recognition protein; IAP = IAP repeat, inhibitor of apoptosis domain; IGG = Immunoglobulin; IMD = Imd pathway; JAST = JAKSTAT; LYS = Lysozyme; ML = MD-2-related lipid recognition; NIM = NIMROD; PGRP = Peptidoglycan recognition protein; PPO = Prophenoloxidase; PRDX = Peroxidase; REL = Relish; SCR = Scavenger receptor; SOD = Superoxide dismutase; SPZ = Spaetzle; SRPN = Serine protease inhibitor; SRRP = Small RNA regulatory pathway; TEP = Thioester-containing protein; TOLL = Toll genes, Toll pathway).

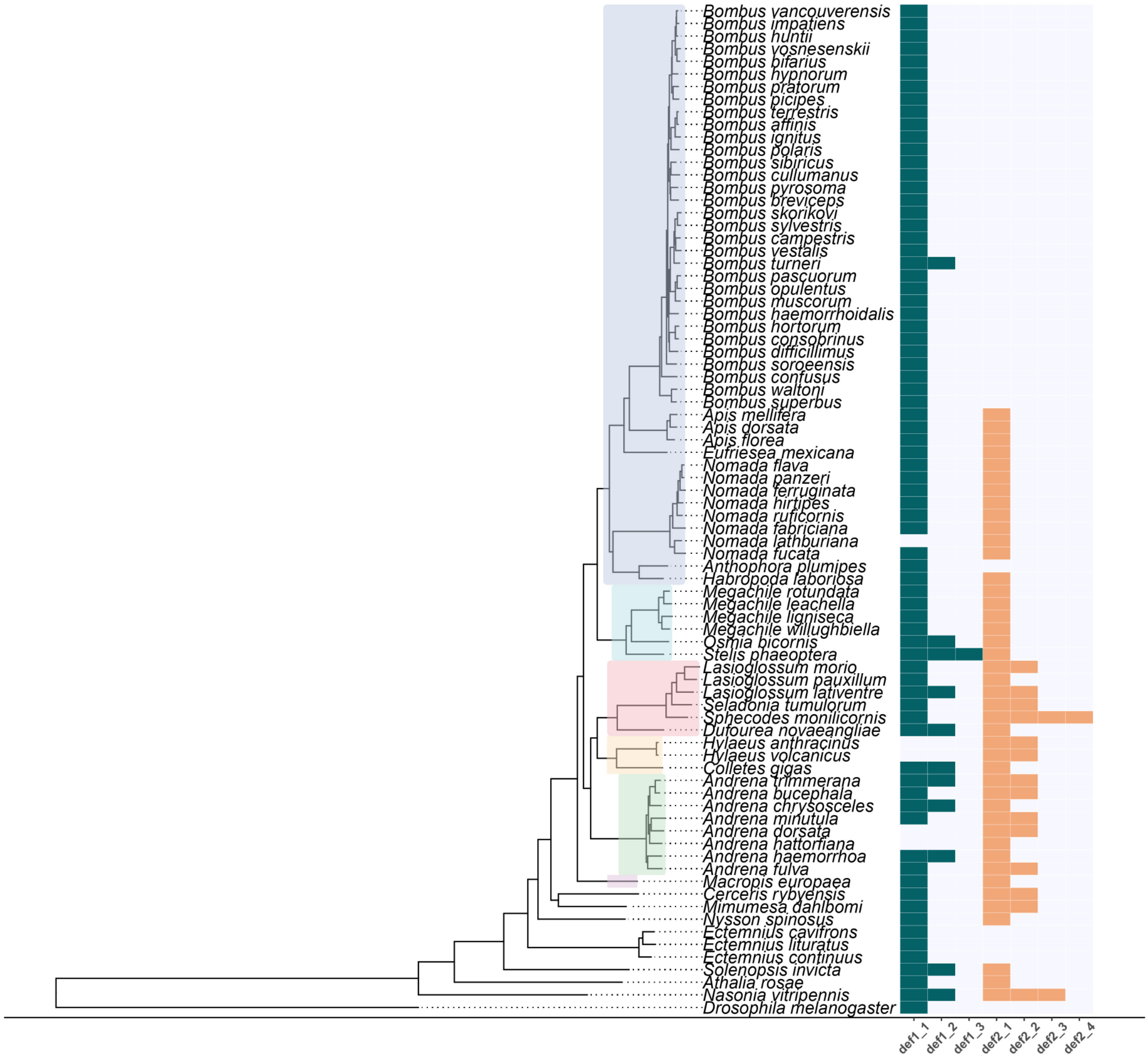

Fig. S4. **Conservation and loss of defensin across bee species.** A combined multi-panel plot displaying: the evolutionary relationships between investigated species are provided in a phylogenetic tree based on a homology-based comparative analysis of predicted proteomes for 80 species, including representatives of six bee families (Apidae = blue; Megachilidae = light blue; Halictidae = pink; Colletidae = light yellow; Andrenidae = light green; Melittidae = light purple); and a heatmap displaying the two defensin gene copies (*Def1* (green) and *Def2* (tan)) in extant bee species, which were identified using homology-based method.

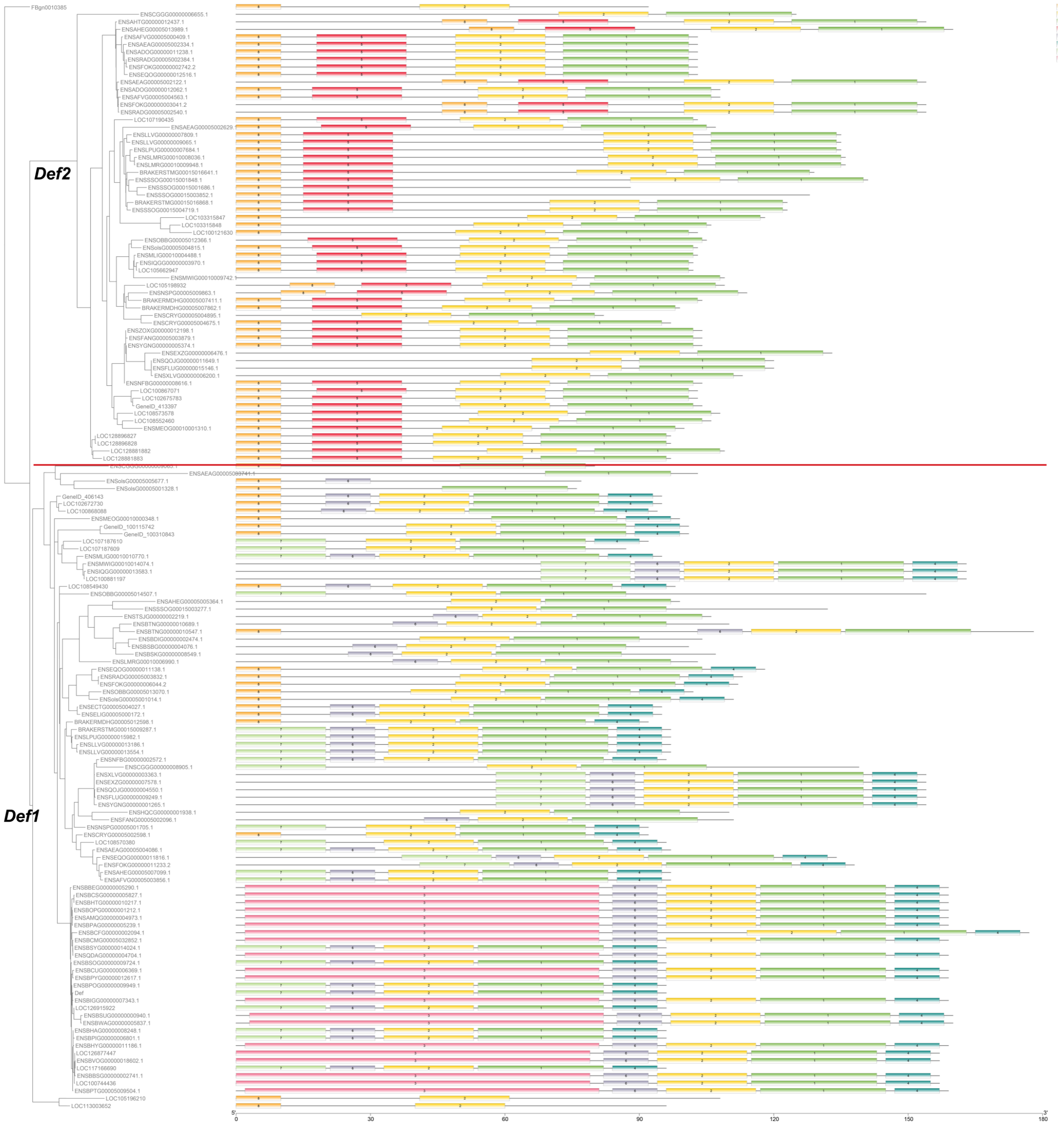

Fig. S5. Conserved and copy-specific protein motifs of defensins across bee species. All defensin gene copies in each species were identified by Orthofinder-based analysis and their gene tree were showing in the left-hand side. Two main copies of *Def1* and *Def2* were separated by a red line. Motifs of each copy of defensin in each species were identified by MEME analysis. Eight different motifs were represented by rectangles with different colours and length.

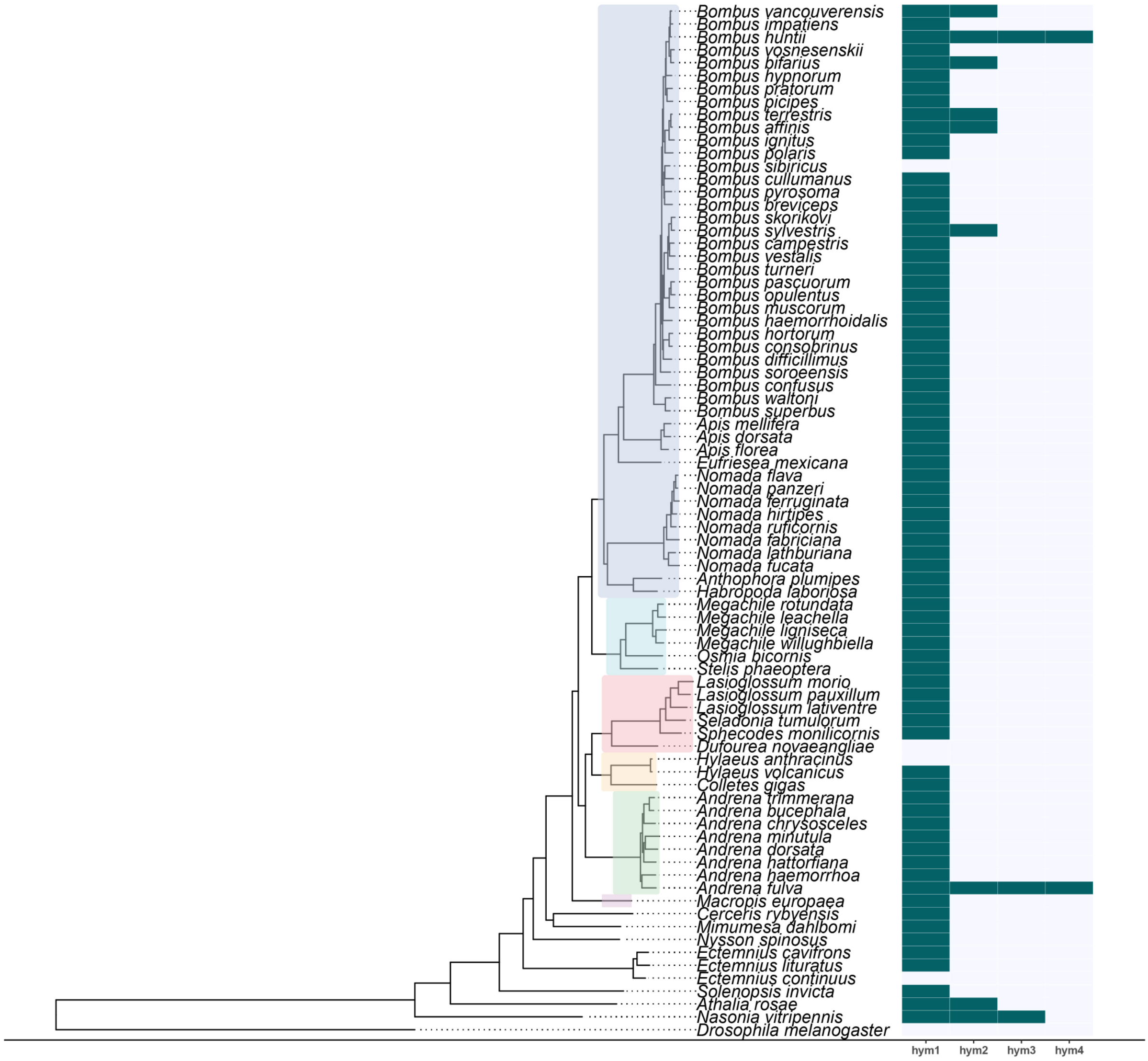

Fig. S6. **Conservation of hymenoptaecin across bee species.** A combined multi-panel plot displaying: the evolutionary relationships between investigated species are provided in a phylogenetic tree based on a homology-based comparative analysis of predicted proteomes for 80 species, including representatives of six bee families (Apidae = blue; Megachilidae = light blue; Halictidae = pink; Colletidae = light yellow; Andrenidae = light green; Melittidae = light purple); and a heatmap displaying correspondingly gene *hymenoptaecin*, which were identified using homology-based method.

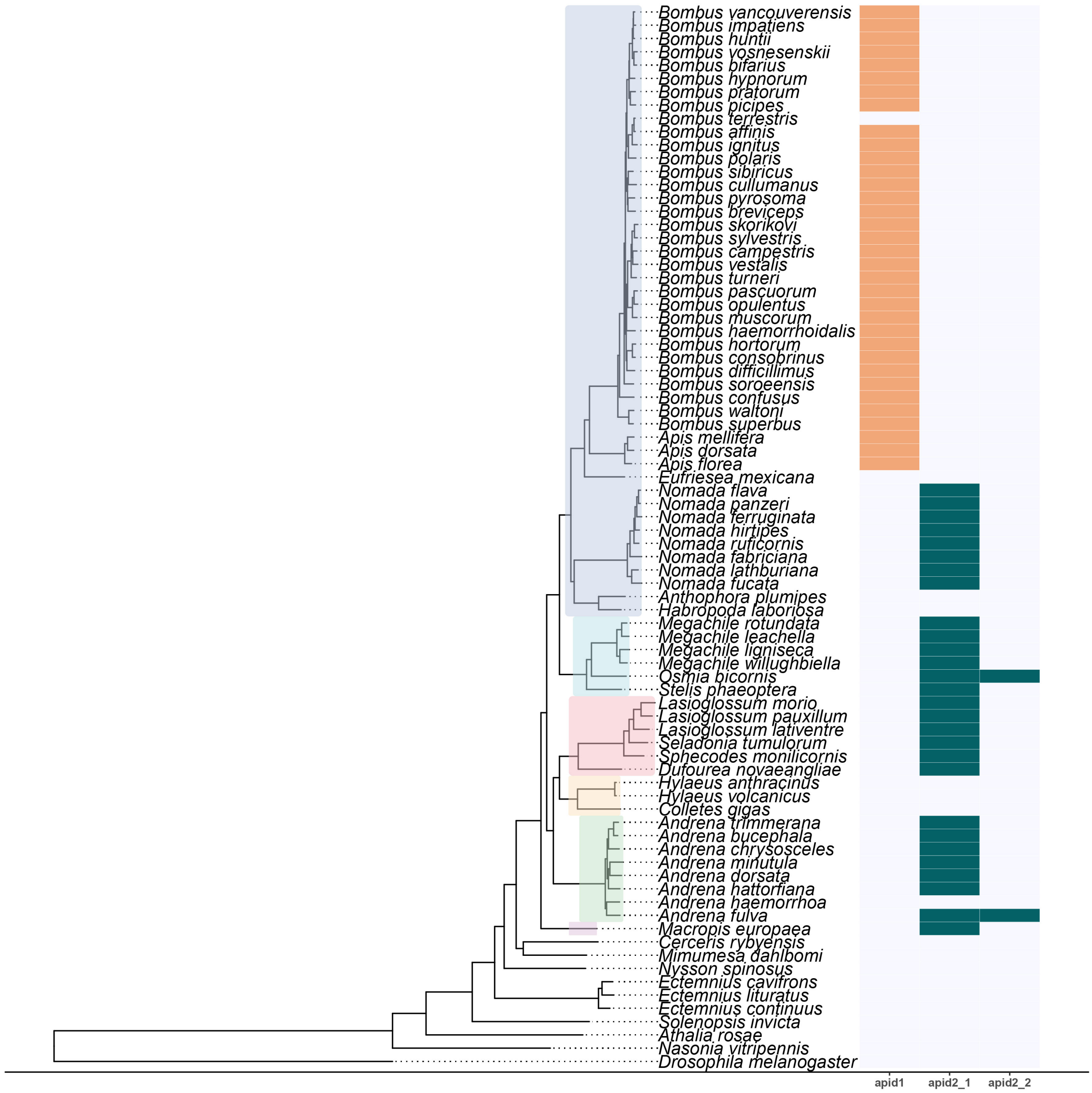

Fig. S7. **Conservation of apidaecin across bee species.** A combined multi-panel plot displaying: the evolutionary relationships between investigated species are provided in a phylogenetic tree based on a homology-based comparative analysis of predicted proteomes for 80 species, including representatives of six bee families (Apidae = blue; Megachilidae = light blue; Halictidae = pink; Colletidae = light yellow; Andrenidae = light green; Melittidae = light purple); and a heatmap displaying correspondingly gene *apidaecin*, which were identified using homology-based method.

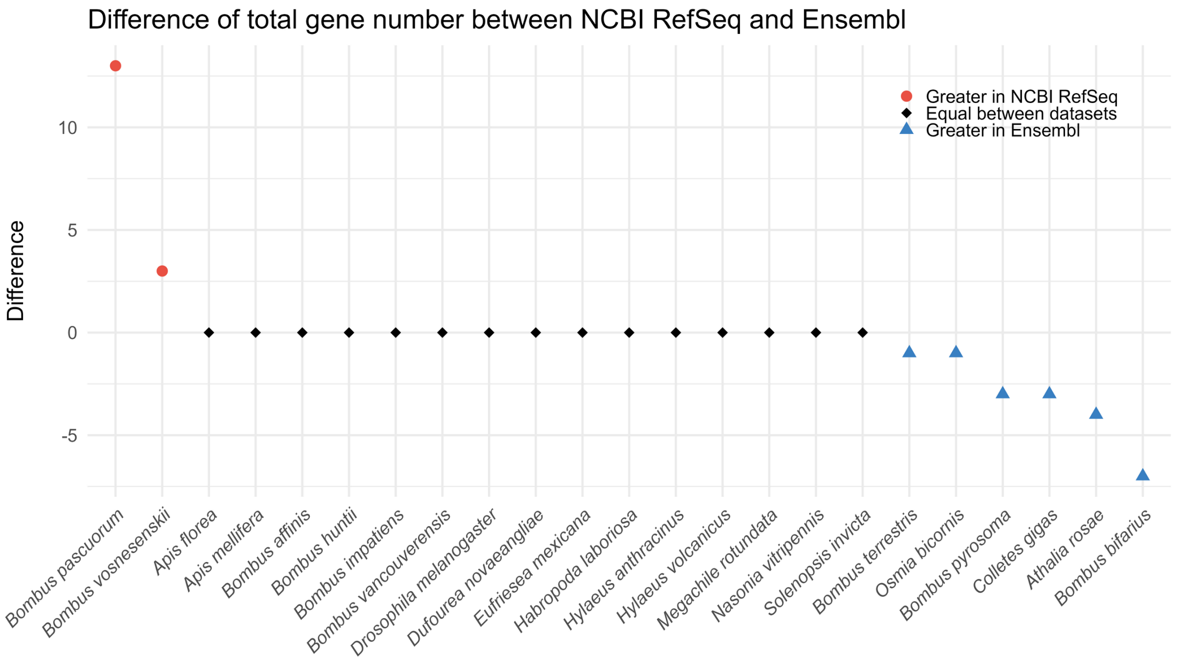

Fig. S8. **Conservation of canonical immune genes in the genome annotations of bee species.** Scatter plot displaying the difference of differentially annotated immune gene number in each of 23 species. Immune genes were identified by Orthofinder-based analysis. The differences were calculated between NCBI RefSeq and Ensembl datasets (number of NCBI RefSeq annotated genes minus number of Ensembl annotated genes). Results of greater-in-NCBI-RefSeq, greater-in-Ensembl, and equal-between-datasets were represented in red circle, black diamond, and blue triangle, respectively.
